# Predictors of atrial fibrillation after embolic stroke of undetermined source in patients with implantable loop recorders

**DOI:** 10.1007/s10072-024-07548-y

**Published:** 2024-04-25

**Authors:** Fabienne Kreimer, Assem Aweimer, Ibrahim El-Battrawy, Adnan Labedi, Ruth Schneider, Arash Haghikia, Andreas Mügge, Michael Gotzmann

**Affiliations:** 1 University Hospital St Josef Hospital, Cardiology and Rhythmology, Ruhr University, Gudrunstraße 56, 44791 Bochum, Germany; 2 University Hospital Bergmannsheil, Cardiology and Angiology, Ruhr University, Bochum, Germany; 3 Department of Molecular and Experimental Cardiology Institut für Forschung und Lehre (IFL), Ruhr University Bochum, Bochum, Germany; 4 University Hospital St Josef Hospital, Neurology, Ruhr University, Bochum, Germany; 5 Department of Cardiology, Angiology and Intensive Care Medicine, Deutsches Herzzentrum der Charité, Berlin, Germany; 6 German Center for Cardiovascular Research (DZHK), Partner Site Berlin, Berlin, Germany; 7 Friede Springer Cardiovascular Prevention Center at Charité, Berlin, Germany

**Keywords:** Embolic stroke of undetermined source, Atrial fibrillation, Age, P-wave parameters, Stroke recurrence

## Abstract

**Background:**

In patients with embolic stroke of undetermined source (ESUS), underlying subclinical atrial fibrillation (AF) is often suspected. Previous studies identifying predictors of AF have been limited in their ability to diagnose episodes of AF. Implantable loop recorders enable prolonged, continuous, and therefore more reliable detection of AF. The aim of this study was to identify clinical and ECG parameters as predictors of AF in ESUS patients with implantable loop recorders.

**Methods:**

101 ESUS patients who received an implantable loop recorder between 2012 and 2020 were included in this study. Patients were followed up regularly on a three-monthly outpatient interval.

**Results:**

During a mean follow-up of 647 ± 385 days, AF was detected in 26 patients (26%). Independent risk factors of AF were age ≥ 60 years (HR 2.753, CI 1.129–6.713, *p* = 0.026), P-wave amplitude in lead II ≤ 0.075 mV (HR 3.751, CI 1.606–8.761, *p* = 0.002), and P-wave duration ≥ 125 ms (HR 4.299, CI 1.844–10.021, *p* < 0.001). In patients without risk factors, the risk of developing AF was 16%. In the presence of one risk factor, the probability increased only slightly to 18%. With two or three risk factors, the risk of AF increased to 70%.

**Conclusion:**

AF was detected in about one in four patients after ESUS in this study. A comprehensive evaluation involving multiple parameters and the existence of multiple risk factors yields the highest predictive accuracy for detecting AF in patients with ESUS.

**Supplementary Information:**

The online version contains supplementary material available at 10.1007/s10072-024-07548-y.

## Introduction

In 20–40% of ischemic strokes, the aetiology initially remains unexplained [1]. Subclinical atrial fibrillation (AF) is often suspected as the underlying cause in patients with an embolic stroke of undetermined source (ESUS). Current guidelines recommend ECG monitoring for at least 24 h after an ischemic stroke to rule out AF. However, the optimal duration and type of monitoring has not yet been conclusively defined [1, 2].

The CRYSTAL AF study demonstrated that ECG monitoring with an implantable loop recorder (ILR) is useful in detecting AF after ESUS [1]. After one year, the AF detection rate in the ILR group was six times higher than in the control group [1]. The recently published LOOP study including patients with a high stroke risk also demonstrated a threefold higher AF detection rate with ILR compared to the control group [3]. In the REVEAL-AF study, the average time between ILR implantation and detection of AF in patients at higher risk of AF was 123 days [4]. Consequently, without ILR, AF would presumably remain undetected in most patients during a shorter observation period.

Current guidelines recommend considering additional, longer-term ECG monitoring with an ILR in selected ESUS patients [2]. Accordingly, an ILR should be implanted in patients who are at high risk of AF based on the presence of cardiovascular risk factors and comorbidities [2]. However, there is limited data on predictors for the detection of AF in ESUS patients with ILR [5, 6].

AF and ESUS may be considered as manifestations of atrial cardiomyopathy characterized by structural, functional, and electrical atrial remodeling [7–9]. Since the P-wave represents the electrical excitation propagation in the atria, P-wave parameters are particularly useful for analyzing and deriving predictors for the presence of atrial cardiomyopathy and manifestation as AF [8, 10]. In the past, studies have identified ECG parameters with an association to later detection of AF [10–12]. However, previous studies on P-wave parameters have a common limitation: the diagnosis of AF was based on symptomatic episodes, incidental documentation of AF in the 12-lead resting ECG or hospitalization for AF [13, 14]. As a result, the validity of the identified P-wave parameters for the occurrence of AF is less precise than with continuous ECG monitoring using ILR, which are able to detect asymptomatic and intermittent, short episodes.

The aim of the present ESUS study was to identify clinical and ECG parameters that were associated with ILR-detected AF during long-term follow-up.

## Methods

This study examined all ESUS patients who underwent ILR implantation between September 2012 and August 2020 at the university hospitals St Josef Hospital and Bergmannsheil Bochum. This study is a subgroup analysis of an ILR study [15]. In the present analysis, we focus exclusively on ESUS as an ILR indication to identify risk factors for AF in an ESUS cohort rather than in a heterogeneous ILR cohort with different indications.

The ILR were manufactured by Medtronic (Reveal DX, Reveal XT, Reveal LINQ), St. Jude Medical (Confirm Rx), and Biotronik (BioMonitor 2-AF, Biomonitor III). Patients provided informed consent, and comprehensive data, including medical history, medication, laboratory results, ECG, and echocardiography parameters, were collected prior to implantation. This study is a retrospective analysis of prospectively obtained data and received approval from the local ethics committee of the Ruhr University Bochum. The study was performed in accordance with the Declaration of Helsinki.

### Inclusion and exclusion criteria, follow-up, and study endpoints

Patients received the ILR within 30 days of ESUS event. All patients with ILR underwent regular examinations at the corresponding hospital ambulatories every three months. Additional outpatient and inpatient visits were available if patients reported symptoms requiring clarification, such as recurrent stroke, transient ischemic attack, or palpitations. Outpatient follow-ups included a review of medical history and an inquiry into ILR data.

The diagnosis of ESUS was made by the neurologists after MRI imaging and exclusion of alternative causes. Only patients with sinus rhythm were included in the analysis, and those with a previous diagnosis of AF were excluded. Patients without any device interrogation reports post-implantation were also excluded.

The primary study endpoint was the first occurrence of AF. Secondary endpoints comprised all-cause death and recurrent ischemic stroke and/or transient ischemic attack. The diagnosis of AF was based on automatic device detection, validated by a cardiologist. Most ILR have a minimum AF detection duration of two minutes. Additionally, each recorded arrhythmia episode and patient-activated episodes were examined. In cases where an episode of ≥ 30 s of irregular heart rhythm, without detectable P-waves, was recorded, the diagnosis of AF was established [1].

All patients received aspirin 100 mg as secondary prophylaxis after ESUS. All patients diagnosed with AF during follow-up received oral anticoagulation instead of aspirin.

Follow-up ended at the latest ILR check, either due to battery depletion, ILR explantation, or the patient discontinuing outpatient follow-up.

### ECG analysis

All patients underwent a comprehensive analysis of the 12-lead ECG recorded within 24 h prior to ILR implantation. The standard 12-lead surface ECG was conducted at a rate of 50 mm/s and a voltage of 10 mm/mV. Two observers who were blinded to the patients' group conducted all evaluations. The ECG analysis focused on P-wave indices and included an assessment of the QRS complex.

The P-wave reflects the atrial depolarization of first the right and then the left atrium. In lead II, the maximum height of the P-wave amplitude was determined. P-wave duration was defined as the maximum duration in any of the 12 leads. P-wave dispersion was calculated by subtracting the minimum P-wave duration from the maximum P-wave duration in the 12-lead ECG [10]. The P-wave axis was determined, with the range of 0° to 75° considered normal [10]. Deviations < 0° were defined as left deviation, and > 75° as right deviation.

**Fig. 1:**
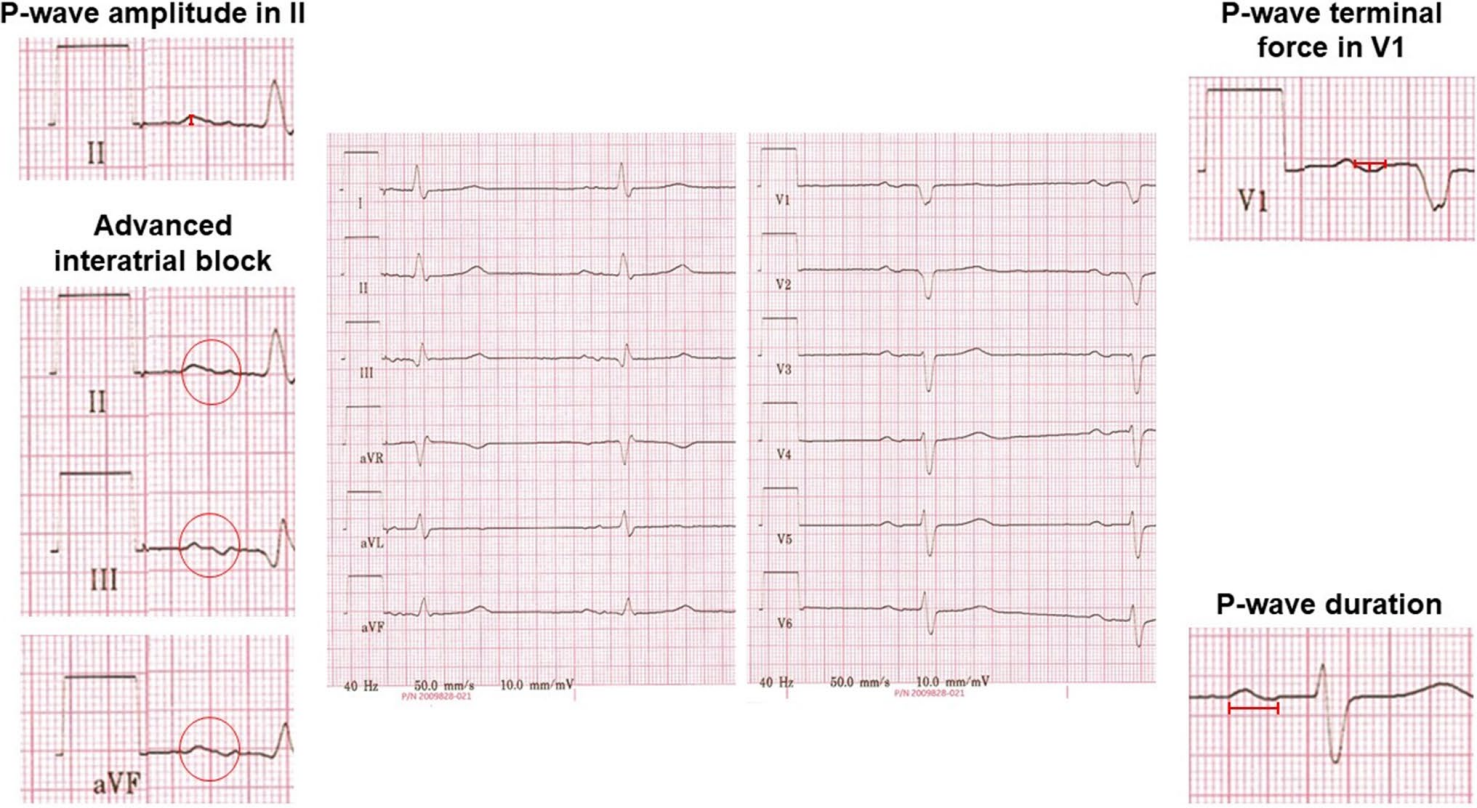
12-lead ECG of a study patient, illustrating several P-wave parameters. An abnormal P-wave amplitude in lead II is assumed at an amplitude < 0.1 mV (top left). An advanced interatrial block is present with a P-wave duration ≥ 120 ms and a biphasic morphology in the inferior leads (bottom left). The P-wave terminal force in lead V1 is calculated by multiplying the duration of the terminal, negative part of the P-wave by the (negative) amplitude of this part and is pathological under -4000 µV*ms (top right). A prolonged P-wave duration is present with a duration > 100 ms and a partial interatrial block from a duration of at least 120 ms (bottom right)

An interatrial block is a block in the interatrial conduction in the Bachmann bundle, causing retrograde excitation of the left atrium. A partial interatrial block was defined as prolonged P-wave ≥ 120 ms, whereas advanced interatrial block was defined as P-wave prolongation ≥ 120 ms, combined with a biphasic morphology in lead III and aVF, and a biphasic or notched morphology in lead II [10]. The P-wave in lead V1 is typically biphasic, with the second, negative term representing left atrial electrical activation. The P-wave terminal force in lead V1 (PTFV1) was calculated by multiplying the depth of the second term by its width (Fig. 1) [10]. QRS complex duration was measured in the lead with the widest QRS complex. The axis of the QRS complex and the T wave were determined, and right and left bundle branch block were defined based on standard criteria.

### Statistics

The numerical values are expressed as mean ± standard deviation. An unpaired t-test (for normally distributed variables) or a Mann–Whitney U-test (for non-normally distributed variables) were used to compare continuous variables between groups. Categorical variables were analyzed using chi-square analysis or Fisher´s exact test. All variables from Tables 1 and 2 were analyzed for an association with the primary study endpoint (AF detection) using a univariate Cox proportional hazard model. All variables that had a significant association with the primary study endpoint (p ≤ 0.05) were submitted to a multivariate Cox model analysis to identify independent predictors of outcome. The following parameters were independently associated with the study endpoint: Age, P-wave amplitude in II, P-wave duration, PTFV1, and advanced interatrial block. Receiver operating characteristic (ROC) analysis was performed on these independent predictors to determine the best cut-off values of the continuous parameters. The ROC curves were used to obtain the cut-off values with the best sensitivity and specificity. The hazard ratio of these variables was determined using a univariate Cox proportional hazard model and is shown in Table 3. To detect a possible multicollinearity of the independent predictors for the occurrence of AF, we used a Pearson correlation analysis.

**Table 1:** Clinical characteristics of the study cohort (*n* = 101)

	Detection of atrial fibrillation (*n* = 26)	No detection of atrial fibrillation (*n* = 75)	*p* value
Age (years)	66.5 ± 9.6	56.3 ± 11.1	< 0.001
Women (♀), *n* (%)	7 (27)	33 (44)	0.125
Body mass index (kg/m^2^)	27.9 ± 3.8	28.3 ± 4.2	0.723
Left ventricular ejection fraction (%)	61.4 ± 3.8	60.1 ± 6.0	0.311
Left atrial diameter (mm)	38.0 ± 6.5	36.5 ± 4.8	0.242
Medical history			
Hypertension, *n* (%)	22 (85)	50 (67)	0.081
Diabetes mellitus, *n* (%)	8 (31)	14 (19)	0.198
Coronary artery disease, *n* (%)	3 (12)	5 (7)	0.421
Labor			
Creatinine (mg/dL)	0.94 ± 0.23	0.87 ± 0.20	0.134
TSH (mIU/L)	1.37 ± 0.76	1.50 ± 0.86	0.523
Medication			
Beta-Blocker, *n* (%)	8 (31)	21 (28)	0.788
ACE-Inhibitors & ARB, n (%)	12 (46)	36 (48)	0.871

*TSH* Thyroid-stimulating hormone; *ARB* Angiotensin II receptor blockers

**Table 2:** ECG parameters of the study cohort (*n* = 101)

	Detection of atrial fibrillation (*n* = 26)	No detection of atrial fibrillation (*n* = 75)	*p* value
Heart rate (beats/min)	67.4 ± 11.3	69.4 ± 10.8	0.401
P-wave amplitude in II (mV)	0.09 ± 0.04	0.13 ± 0.04	0.001
P-wave duration (ms)	113 ± 30	103 ± 17	0.038
P-wave dispersion (ms)	20 ± 12	20 ± 8	1.000
P-wave axis (°)	51 ± 38	51 ± 16	0.969
P-wave right axis deviation, *n* (%)	2 (8)	2 (3)	0.297
P-wave left axis deviation, *n* (%)	2 (8)	1 (1)	0.178
P-wave terminal force in V1 (µV*ms)	−4125 ± 2407	−3480 ± 2085	0.195
Abnormal P-wave terminal force in V1 (µV*ms)*	14 (54)	23 (31)	0.035
Partial interatrial block, *n* (%)	12 (46)	17 (23)	0.023
Advanced interatrial block, *n* (%)	4 (15)	0 (0)	0.004
PR interval (ms)	183 ± 36	174 ± 27	0.178
QRS duration (ms)	96 ± 20	89 ± 13	0.052
QRS axis (°)	15 ± 29	29 ± 33	0.060
Right bundle branch block, *n* (%)	2 (8)	1 (1)	0.162
Left bundle branch block, *n* (%)	1 (4)	2 (3)	1.000
T-wave axis (°)	46 ± 41	40 ± 26	0.414

^*^ defined as ≤—4000 µV*ms

**Table 3:** Univariate and multivariate analysis for identifying risk factors of atrial fibrillation detection

	HR	95% CI	*p* value	HR	95% CI	*p* value
Age ≥ 60 years	3.180	1.335–7.570	0.009	2.753	1.129–6.713	0.026
P-wave amplitude in II ≤ 0.075 mV	2.884	1.322–6.290	0.008	3.751	1.606–8.761	0.002
P-wave duration ≥ 125 ms	4.309	1.938–9.580	< 0.001	4.299	1.844–10.021	< 0.001
Abnormal PTFV1 (µV*ms)*	2.184	1.008–4.735	0.048			
Advanced interatrial block	11.491	3.819–34.571	< 0.001			

*CI* Confidence interval; *HR* Hazard ratio; *PTFV1* P-wave terminal force in V1; * defined as ≤—4000 µV*ms

Freedom from AF was assessed using the Kaplan–Meier method, with log-rank curve comparisons. Independent predictors identified by the multivariate Cox proportional hazard survival model were used to derive a prognostic score to categorize patients into different risk groups. Results are presented as hazard risk, and a *p*-value < 0.05 was considered significant. All probability values reported are two-sided.

## Results

In both neurology clinics, there were 5387 patients with an ischemic stroke in the inclusion period between 2012 and 2020, 513 of whom had an ESUS. Of these, a total of 106 patients received an ILR, 101 patients were finally included in this study, as five patients could not be followed up. The mean age of the cohort at the time of implantation was 58.9 ± 10.7 years and 40 patients were female (39.6%). Seventy-two patients (71.3%) were diagnosed with arterial hypertension, 22 patients (21.8%) with diabetes mellitus, and 8 patients (7.9%) with coronary artery disease. The mean left ventricular ejection fraction was 60.4% ± 5.4% and left atrial diameter 36.9 mm ± 5.2 mm. The patients received the following devices: Medtronic (Reveal DX [*n* = 2], Reveal XT [*n* = 25], Reveal LINQ [*n* = 63]), St. Jude Medical (Confirm Rx [*n* = 5]), and Biotronik (BioMonitor 2-AF [*n* = 4], Biomonitor III [*n* = 2]).

### Follow-up, patient characteristics, and ECG analysis

**Fig. 2:**
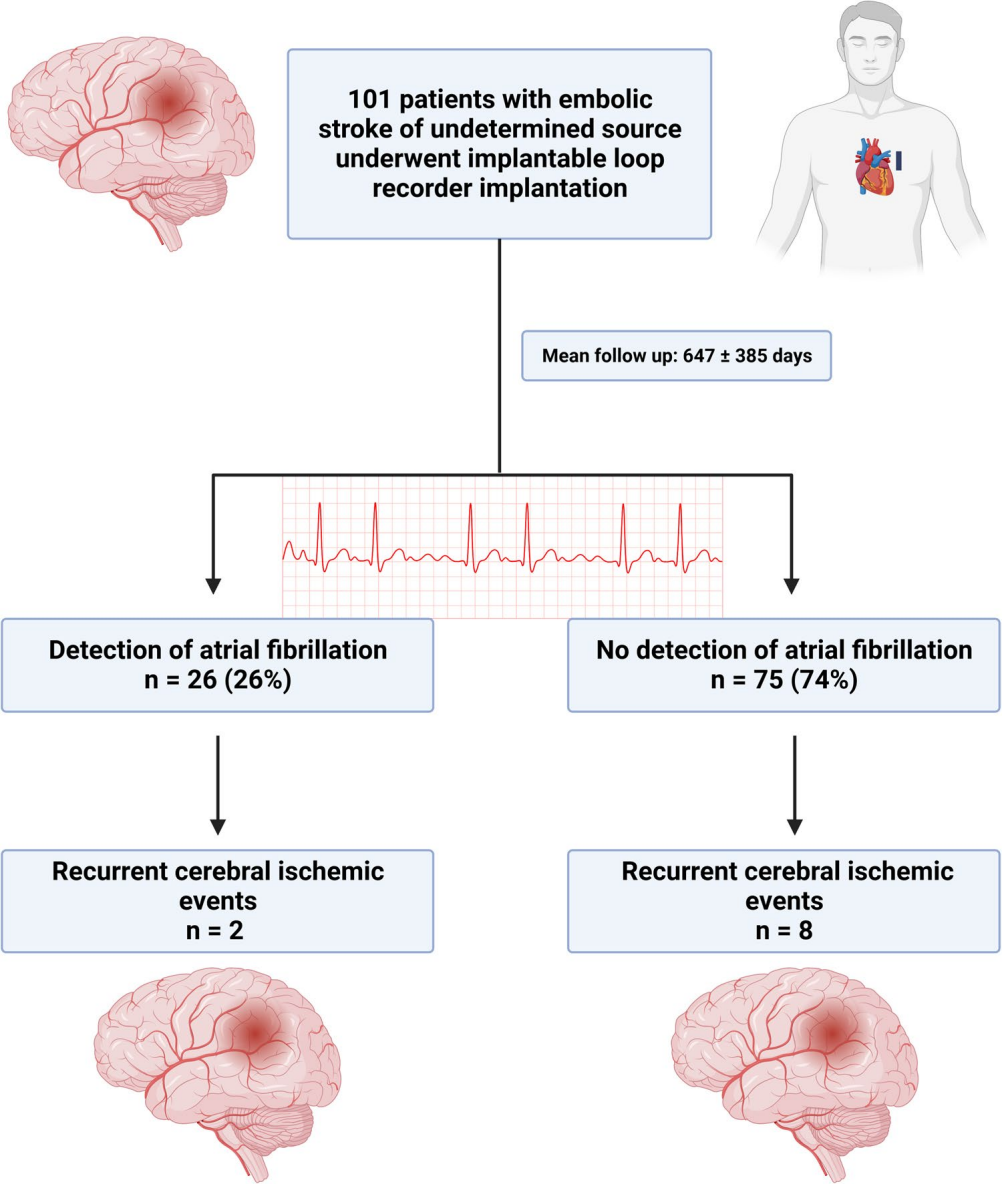
Flow chart presenting the outcome of the ESUS cohort

The mean follow-up time was 647 ± 385 days. Twenty-six patients (26%) were diagnosed with AF during the observation period based on loop recorder analysis (Fig. 2). The diagnosis of AF was established after a mean of 231 ± 196 days (minimum 17 days, maximum 760 days).

Patients with AF were significantly older (66.5 ± 9.6 years vs. 56.3 ± 11.1 years, *p* < 0.001). All other clinical characteristics of the study cohort revealed no association with AF (Table 1).

Several abnormal P-wave parameters were significantly associated with the detection of AF: P-wave amplitude in lead II (0.09 ± 0.04 mV vs. 0.13 ± 0.04 mV, *p* = 0.001), P-wave duration (113 ± 30 ms vs. 103 ± 17 ms, *p* = 0.038), partial interatrial block (46% vs. 23%, *p* = 0.023), advanced interatrial block (15% vs. 0%, *p* = 0.004), and abnormal PTFV1 (54% vs. 31%, *p* = 0.035). In contrast, ECG parameters of ventricular depolarization and repolarization exhibited no association with the occurrence of AF (Table 2).

### Predictors of atrial fibrillation, and risk score

On univariate Cox analysis, age, p-wave amplitude in lead II, P-wave duration, abnormal PTFV1, and advanced interatrial block were significantly associated with the primary study end point (Table 3).

Using receiver operating characteristic analysis, cutoff values for separating the study cohort were age ≥ 60 years (Area under the curve [AUC] 0.761, *p* < 0.001), P-wave amplitude in lead II ≤ 0.075 mV (AUC 0.685, *p* = 0.005), and P-wave duration ≥ 125 ms (AUC 0.593, *p* = 0.161).

Multivariate analysis identified age ≥ 60 years, P-wave amplitude in lead II ≤ 0.075 mV, and P-wave duration ≥ 125 ms as independent predictors of AF occurrence (Table 3). The Pearson correlation analysis revealed no correlations between age and P-wave duration, no correlations between P-wave amplitude in lead II and P-wave duration and only a weak correlation between age and P-wave amplitude in lead II (r =  − 0.284, *p* = 0.004). A multicollinearity between the three independent risk factors could therefore be excluded.

**Fig. 3:**
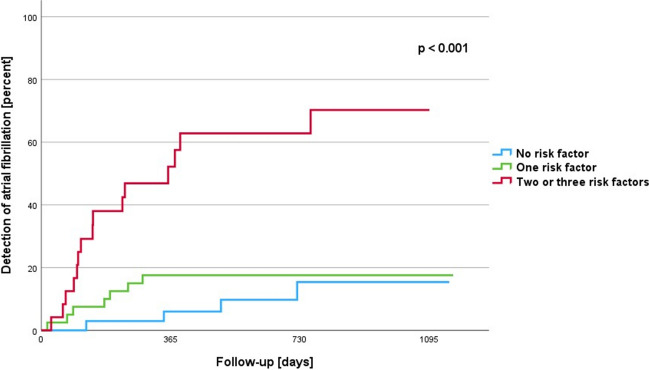
Kaplan–Meier curves presenting the risk of AF detection depending on the number of independent risk factors (age ≥ 60 years, P-wave amplitude in lead II ≤ 0.075 mV, and P-wave duration ≥ 125 ms)

A predictive model that divided the study cohort in patients with lower to high risk of AF occurrence based on these three independent predictors. Patients without a risk factor had a 16% risk of AF occurrence. If one risk factor was present, the risk was 18%. The risk of AF increased to 70% with two or three risk factors. One- and two-year occurrence rates were 7% and 10% when no risk factor was present, 18% in the presence of one risk factor, and 52% and 63% in the presence of two or three risk factors (Fig. 3, Supplemental Fig. 1).

### All-cause death, and recurrent ischemic cerebral events

During follow-up, no patient died. Ten patients had a recurrent ischemic stroke or transient ischemic attack: nine patients had ischemic stroke events and one patient suffered a transient ischemic attack (Fig. 2). According to the TOAST classification, two of these events were of cardioembolic origin, one was of microangiopathic origin, one was of macroangiopathic origin, three were recurrent ESUS and in three events the underlying cause could not be determined. Two patients with a recurrent stroke were diagnosed with AF (20%). In one patient AF was detected three months before the recurrent stroke and he received apixaban (2 × 5 mg daily) since AF detection without discontinuation, in the other patient AF was diagnosed during hospitalisation for the recurrent stroke and he only received antiplatelet therapy with aspirin (100 mg daily) until this second event.

All parameters from Tables 1 and 2 were analyzed for an association with stroke recurrence. Clinical parameters with an association to recurrent ischemic cerebral events were diabetes mellitus (50% vs. 19%, *p* = 0.023) and Creatinine (1.02 ± 0.25 mg/dl vs. 0.87 ± 0.20 mg/dl, *p* = 0.036). AF detection was not associated with the occurrence of recurrent ischemic cerebral events (20% vs. 26%, *p* = 1.000). P-wave parameters also demonstrated no association with recurrent ischemic stroke events.

## Discussion

In the present study, ECG parameters and clinical factors for the prediction of AF were investigated in an ESUS cohort. Twenty-six patients (26%) were diagnosed with AF during follow-up, which is consistent with previous studies that reported AF detection rates after ESUS of 12% to 41% [16–23]. However, in this study, the sample size was small und the overall event rate was low. The randomized, controlled CRSTAL AF study involving 441 patients aimed to compare the effectiveness of long-term monitoring with an ILR against conventional follow-up (control) in detecting AF among individuals with cryptogenic stroke [1]. By twelve months, AF was detected in 12.4% of patients in the ILR group versus 2.0% in the control group (*p* < 0.001) [1].

The combination of the independent risk factors (age ≥ 60 years, P-wave amplitude in lead II ≤ 0.075 mV, and P-wave duration ≥ 125 ms) in a predictive model for AF detection was suitable to divide our study cohort into patients with lower to high risk of AF detection.

An advantage of this study is the long follow-up period of 647 ± 385 days, providing a longer observation time compared to previous studies [17, 19, 22, 24, 25].

Older age is a major risk factor for AF [2]. Due to the increasing life expectancy of the population, it is assumed that the prevalence of AF will increase two- to threefold in the next years [2]. Older age is also associated with an increased risk of ischemic stroke [26]. In our ESUS cohort, patients with detected AF were significantly older than patients without detected AF. In addition, age ≥ 60 years was an independent risk factor for the occurrence of AF. These results are consistent with previous studies, all of which demonstrated an increased risk of AF detection in ESUS patients with increasing age [16, 17, 20, 22, 23, 27, 28].

Atrial cardiomyopathy includes structural remodeling of the atria, e.g., increased fibrosis and left atrial enlargement. In the past, it has been described that ESUS patients have an increased left atrial volume compared to healthy, age- and sex-matched individuals [29]. In addition, previous studies have demonstrated that left atrial enlargement in ESUS patients is also associated with an increased risk of AF [16, 25, 27]. However, this association may be weak, as left atrial enlargement is rarely an independent predictor of AF detection [20]. In addition, a recently published study also failed to identify a correlation between a higher left atrial volume index and the detection of AF after ESUS [23].

This study also revealed no difference in left atrial diameter between patients with and without AF. Furthermore, our ESUS cohort presented with on average medium-normal values of left atrial diameter. However, it is conceivable that other parameters of left atrial function and morphology may be predictors for the occurrence of AF [7, 8, 16, 25, 27]. Unfortunately, in this study, only the diameter of the left atrium could be analyzed.

The P-wave represents the propagation of electrical excitation within the atria [10]. The morphology and length of the P-wave are influenced by various factors, including atrial size, fibrosis, and intra- or interatrial conduction disorders [10]. In this study, several P-wave parameters were linked to the detection of AF following an ESUS: P-wave amplitude in lead II, P-wave duration, PTFV1, as well as partial and advanced interatrial blocks. Notably, patients with ILR were studied, which allows a more reliable diagnosis of AF and consequently a more reliable classification of the ESUS cohort into patients with and without AF, thereby increasing the overall validity of P-wave parameters as predictors of AF.

In a prospective study involving 236 patients diagnosed with ESUS, an ILR was implanted during the index hospitalization [21]. The study assessed pre-specified variables, including CHA_2_DS_2_-VASc, P-wave duration, P-wave morphology, premature atrial beats within 24 h, supraventricular tachycardia within 24 h, left atrial end-systolic volume index, Troponin-T, NT-proBNP, and D-dimer [21]. Subsequently, 84 patients (36%) were found to have subclinical AF [21]. In univariate analysis, all pre-specified variables showed a significant association with AF detection [21]. However, in multivariate analysis, only premature atrial beats within 24 h, P-wave duration, P-wave morphology, and left atrial end-systolic volume index remained significant predictors of AF [21].

In addition to P-wave duration, previous studies have also identified partial and advanced interatrial block, P-wave dispersion and abnormal PTFV1 as predictors of AF after ESUS [24, 25, 29, 30]. In particular, advanced interatrial block and abnormal PTFV1 appear to have a high prognostic effect [10]. Similarly, this study revealed a high prevalence of abnormal PTFV1 and partial interatrial block and a significant association with AF detection. However, none of the parameters was an independent predictor in the multivariate analysis. Partial interatrial block is defined as prolonged P-wave duration ≥ 120 ms and in this analysis a P-wave duration of ≥ 125 ms was an independent risk factor for AF, which may indicate that a higher threshold is required in a cohort with an increased prevalence of P-wave abnormalities.

Advanced interatrial block is strongly associated with AF [8, 10, 24]. The reason it was not an independent predictor of AF detection in this study may have been the relatively small cohort and low prevalence.

Abnormal PTFV1 can be understood as an expression of left atrial enlargement, although probably only a rather weak association, as it could also be caused by interatrial conduction delay. Nevertheless, this could be a reason why abnormal PTFV1 was not an independent risk factor for AF in this ESUS cohort, as the patients had medium to high normal left atrial diameter values and there was no difference between the groups with and without AF.

The presence of atrial cardiomyopathy is heterogeneously defined in studies [8]. An example of a common study definition is found in the ARCADIA study, which compares aspirin to apixaban in patients with ESUS and considers atrial cardiomyopathy as the presence of at least one of the following parameters: PTFV1 > 5000 µV × ms, NT-proBNP > 250 pg/mL, and/or indexed left atrial diameter > 3 cm/m^2^ [31]. In a prospective study involving 183 patients with ischemic stroke, these criteria were applied, leading to the detection of atrial cardiomyopathy in 57% of the patients [27]. After a six-month follow-up, AF was detected in 33% of patients with atrial cardiomyopathy compared to 14% of patients without atrial cardiomyopathy (*p* = 0.003) [27]. Notably, atrial cardiomyopathy did not emerge as an independent risk factor for detecting AF following ischemic stroke [27]. A reason for this could be that atrial cardiomyopathy was defined based on the presence of one parameter instead of a combination of several parameters, e.g., elevated NT-proBNP levels without additional atrial-specific values, thus encompass a more heterogeneous cohort.

The findings of a recently published study support this direction, as it demonstrated that the presence of three parameters identified as risk factors for AF (abnormal PTFV1, left atrial end-systolic indexed volume > 34 ml/m^2^, and BMI > 25 kg/m^2^) provided the highest predictive probability [25].

It suggests that a multiparametric evaluation is superior, both in determining the presence of atrial cardiomyopathy in ESUS patients and in predicting the probability of AF detection, compared to single parameters. This is also reflected in the AF risk score in this study, as the risk of AF detection was significantly higher with at least two risk factors compared to none or only one risk factor.

## Limitations

The main limitation of this study is its retrospective nature. In addition, the study cohort is relatively small, and the AF detection rate was low, although comparable to other studies, which may lead to difficulties in detecting statistically significant differences, and which could consequently limit the validity of some comparisons.

Detection of subclinical AF in patients with ESUS suggests that AF caused the ischemic stroke. However, a clear causality cannot be derived, particularly if a long time has passed since the first detection of AF [32–34]. In addition, patients with ESUS might have had more than one possible source of embolism [35].

In addition, not all ESUS patients from both clinics were included, only those who received an ILR, which could have resulted in a selection bias. This study could not compare ESUS patients with and without ILR. Patients who did not receive ILR may have been older, could not be followed up, or had more lifetime-limiting comorbidities.

Furthermore, in-depth echocardiography and the analysis of laboratory markers (such as B-type natriuretic peptide and troponin) could have enhanced predictive accuracy.

## Conclusion

The strength of this study is the identification of multiple P-wave parameters that were associated with the detection of AF in ESUS patients who received long-term and continuous monitoring by ILR, thereby increasing the validity of the identified predictors. Multi-parametric assessment and the presence of multiple risk factors provide the best predictive accuracy for AF detection in ESUS patients and may help to identify those who would benefit most from ILR and closer follow-up. In the future, randomized controlled trials and large ESUS registry studies will be needed to identify risk factors more accurately for first ESUS events and recurrent strokes to develop prediction models. In addition, experimental and translational studies are needed to investigate ESUS pathways.

## Supplementary Information

Below is the link to the electronic supplementary material.

Supplementary file1 (DOCX 123 kb)

## Data Availability

The data are available from the corresponding author on reasonable request.

## Abbreviations

AF: Atrial fibrillation
ESUS: Embolic stroke of undetermined source
ILR: Implantable loop recorder
