# Supplementary material for: Predictors of atrial fibrillation after embolic stroke of undetermined source in patients with implantable loop recorders
Source: Neurol Sci. 2024 Apr 25;45(10):4903–12. doi: 10.1007/s10072-024-07548-y (PMC11422254; doi:10.1007/s10072-024-07548-y)

**Sup. Fig. 1** Kaplan-Meier curves presenting the risk of AF detection depending on the combination of the identified risk factors.


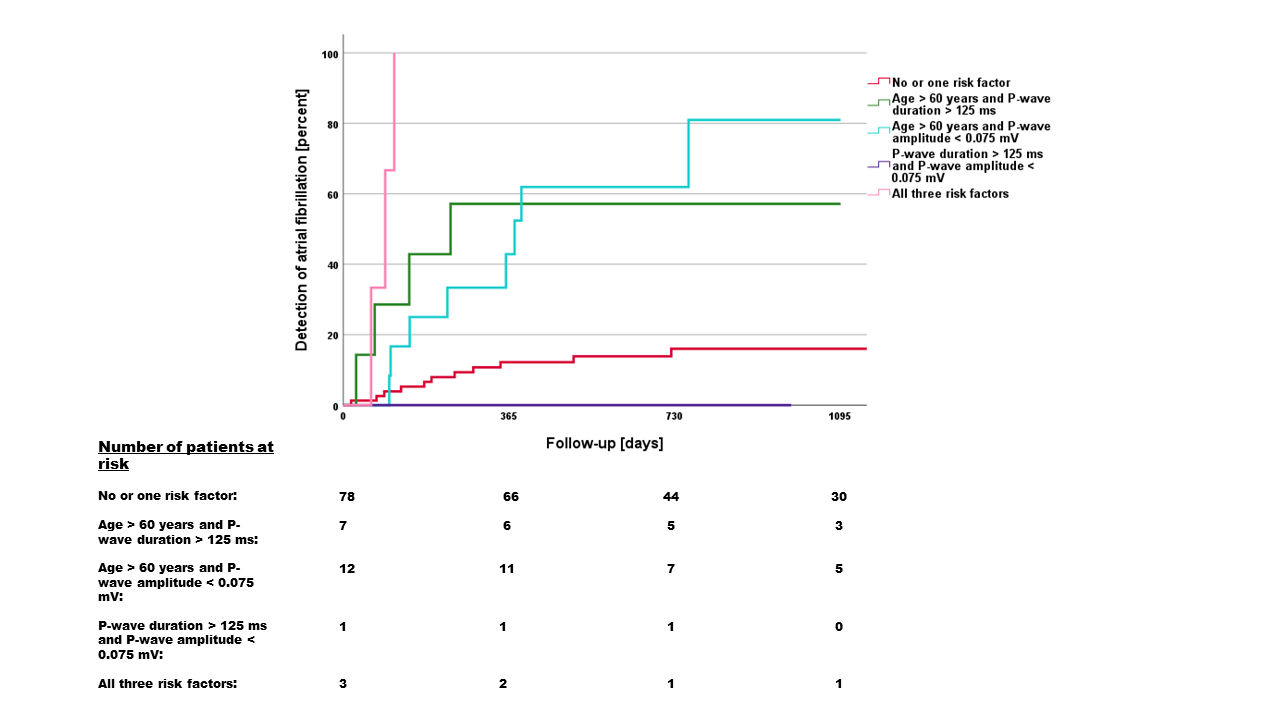

Supplement: Supplementary file 1 — Supplementary file1 (DOCX 123 kb) [file 10072_2024_7548_MOESM1_ESM.docx]
